# Spectrum of *FGFR2* Mutations and Plausible Phenotypic Modifiers Contributing to the Etiology of Syndromic Craniosynostosis in Sub‐Saharan Africans

**DOI:** 10.1155/ijog/5753150

**Published:** 2026-11-11

**Authors:** Lord Jephthah Joojo Gowans, Mavis Dansowaa Asante, Daniel Kwesi Sabbah, Gideon Okyere Mensah, Bruce Tsri, Christian Opoku Asamoah, Joseph Believe Dordor, Solomon Obiri-Yeboah, Tamara Busch, Azeez Butali, Peter Donkor, Michael Lawrence Cunningham

**Affiliations:** ^1^ Department of Biochemistry and Biotechnology Kwame Nkrumah University of Science and Technology Kumasi Ghana knust.edu.gh; ^2^ National Cleft Care Center Komfo Anokye Teaching Hospital Kumasi Ghana kathhsp.org; ^3^ School of Dentistry Kwame Nkrumah University of Science and Technology Kumasi Ghana knust.edu.gh; ^4^ Department of Oral Pathology Radiology and Medicine The University of Iowa Iowa City Iowa USA uiowa.edu; ^5^ Iowa Institute of Oral Health Research University of Iowa Iowa City Iowa USA uiowa.edu; ^6^ Department of Surgery School of Medical Sciences Kwame Nkrumah University of Science and Technology Kumasi Ghana knust.edu.gh; ^7^ Division of Craniofacial Medicine Department of Pediatrics University of Washington Seattle Washington USA washington.edu; ^8^ Center for Developmental Biology and Regenerative Medicine Seattle Children′s Research Institute Seattle Washington USA

**Keywords:** craniosynostosis, *FGFR2*, genetic modifiers, genetic syndromes, Ghanaian population, whole exome sequencing

## Abstract

Craniosynostosis, the premature fusion of cranial sutures resulting from disrupted molecular and cellular mechanisms of suture patency, ranks as the second most common craniofacial birth defect. It manifests as syndromic (15%–30%) or nonsyndromic (70%–85%) forms and has multifactorial etiology. This cross‐sectional, observational, family‐based study is aimed at elucidating the genetic basis of syndromic craniosynostosis in a Ghanaian cohort presenting with characteristic phenotypes of *FGFR2*‐related syndromes. Eight case–parent trios with syndromic craniosynostosis were recruited, and saliva/buccal swab samples were collected for DNA extraction. Whole exome sequencing (WES) of DNA samples was performed on the Illumina HiSeq platform at 100x read depth in a paired‐end configuration. Quality control, read alignment, and variant calling followed the Sentieon V202112.01 workflow, with variant annotation utilizing Ensembl VEP V104. Variants were filtered for minor allele frequency < 0.01 across databases, and pathogenicity was evaluated using 11 dbNSFP‐embedded tools and SpliceAI. ClinVar and LOVD were used to verify the pathogenicity of variants. Gene expression, enrichment and interaction analyses, and associated phenotype ascertainment utilized various databases such as DECIPHER and OMIM. Five probands harbored de novo pathogenic variants in *FGFR2* (p.Asp336Gly, p.Cys342Ser, p.Pro253Arg, p.Gly272del, and p.Ser252Trp). However, lack of paternal samples limited the determination of the de novo status of two variants (p.Ser252Trp and p.Cys342Gly) in three probands. These variants affected multiple cranial suture types, including coronal, sagittal, metopic, and lambdoid, and were associated with the clinical diagnoses of Apert and Crouzon syndromes. Additional likely pathogenic variants in other genes that may plausibly contribute to phenotypic variability were also observed. In silico functional analysis buttressed the role of *FGFR2* and plausible variants contributing to phenotypic variability. Thus, *FGFR2* variants contribute to syndromic craniosynostosis among Ghanaians, with additional genetic variants plausibly influencing phenotypic variability among probands. This study may impact genetic counseling (prenatal testing and recurrence risk) and other clinical management of craniosynostosis.

## 1. Introduction

Craniosynostosis (OMIM#123100) is the most common craniofacial anomaly after orofacial clefts (OFCs), with an incidence of about 1 in 2000–2500 live births [1, 2]. The condition arises from the premature fusion of cranial sutures (sagittal, metopic, coronal, and lambdoid), resulting from the failure of molecular and cellular processes that maintain suture patency. As a result, brain growth during infancy and adolescence may be limited due to skull deformation [3]. Severe complications, including facial and head deformity (scaphocephaly, trigonocephaly, brachycephaly, and plagiocephaly), blindness, hearing loss, and dental and intellectual disability, may result from craniosynostosis [4]. It thus imposes a significant burden on the physical health, physiology, psychosocial functioning, and economic status of affected individuals and families [5, 6].

Craniosynostosis may occur as a clinical presentation of a genetic syndrome (15%–30% of cases) or be nonsyndromic, occurring without other deformities [7]. Syndromic craniosynostosis, which co‐occurs with other anomalies, including cranial base ossification, midface hypoplasia, and limb anomalies, usually results from a genetic predisposition [8]. Implicated syndromes include Carpenter, Apert, Crouzon, Pfeiffer, and Saethre–Chotzen syndromes [1]. Both syndromic and nonsyndromic craniosynostosis emanate from variants in 57–79 genes, including *FGFR1–3*, *MSX2*, *TWIST1*, *FBN1*, *EFNB1*, *RECQL4*, *RAB23*, *ERF*, and *TGFBR1–2* [3, 5, 9]. Among the genes implicated in the etiology of syndromic craniosynostosis, pathogenic variants in fibroblast growth factor receptors (FGFRs) 1–3 (*FGFR1*, *FGFR2*, and *FGFR3*) account for most syndromic presentations, leading to syndromes such as Pfeiffer, Apert, Crouzon, and Muenke [7, 10]. FGFRs encode transmembrane tyrosine kinase receptors comprising three domains, including extracellular immunoglobulin‐like domains, a transmembrane domain, and an intracellular tyrosine kinase domain. The overwhelming majority of reported pathogenic FGFR variants implicated in syndromic craniosynostosis are heterozygous gain‐of‐function (GoF) missense variants. Such GoF variants constitutively activate downstream signaling, culminating in premature osteoblast differentiation and suture fusion, which are the hallmarks of craniosynostosis [10].

The genotype–phenotype correlation for FGFRs is largely specific. Pfeiffer syndrome may result from pathogenic variants in either *FGFR1* (often p.Pro252Arg) or *FGFR2* [11]. Apert syndrome arises almost exclusively from two adjacent Exon IIIa variants in *FGFR2* (p.Ser252Trp and p.Pro253Arg), with p.Ser252Trp accounting for most cases (~66%) and p.Pro253Arg accounting for the remainder [12]. Crouzon syndrome is characterized by a broader spectrum of pathogenic *FGFR2* variants clustered in the IgIIIa/IIIc region [13]. Muenke syndrome is causally linked to a single recurrent *FGFR3* variant, p.Pro250Arg [14]. Although this clustering of pathogenic mutations may suggest mutational hotspots, it also reflects strong positive selection during spermatogenesis, that is, a paternal age effect [10].

Interestingly, irrespective of the relatively strong genotype–phenotype correlation, FGFR‐related craniosynostosis syndromes exhibit striking phenotypic heterogeneity, even among individuals harboring the same variant. Variable penetrance and expressivity are common in Muenke syndrome cases harboring the same p.Pro250Arg variant in *FGFR3*, with some affected individuals presenting with severe bicoronal craniosynostosis and developmental delay, whereas others are clinically silent carriers, even within the same family [14]. In *FGFR1*‐related Pfeiffer syndrome, variable intrafamilial penetrance and expressivity have been documented, with carriers of an identical pathogenic variant (p.Cys277Tyr) presenting with severe, mild, or subclinical phenotypes [11]. For *FGFR2*‐related Crouzon syndrome, cascade testing as a sequel to molecular diagnosis of a severely affected proband demonstrated that mildly affected parents and grandparents also carried the same splice‐site variant, c.1032G>A [13]. In Apert syndrome, the ostensibly tight genotype–phenotype correlation for the p.Ser252Trp variant has recently been challenged by a reported case presenting with unicoronal synostosis and near‐absent syndactyly, which is atypical for this well‐characterized variant [12]. It is hypothesized that the variable penetrance and expressivity observed in FGFR‐related craniosynostosis syndromes may emanate from modifier genes, epigenetic factors, somatic mosaicism, and stochastic developmental noise [7].

Irrespective of the advances in understanding the genetic basis of craniosynostosis, most studies have focused predominantly on populations of European and Asian ancestry. Ostensibly, almost all the landmark genotype–phenotype studies of FGFR‐related craniosynostosis were conducted in European, North American, and East Asian cohorts. Notably, there is a paucity of genetic studies in African populations, including Ghana. A recent systematic review and meta‐analysis on craniosynostosis across eight African countries identified only 14 case reports (27 cases) and 14 prospective/retrospective studies (620 patients). Syndromic craniosynostosis accounted for ~25% of the cases but had minimal molecular characterization [15]. This suggests a crucial gap in craniosynostosis diagnostic capacity (including limited access to molecular/genetic testing) in Africa and other low‐ and middle‐income countries. This observation may lead to under‐ascertainment and misclassification of FGFR‐related syndromes in African settings [16]. This observation recapitulates the broader underrepresentation of Africans in genomics research [17]. In essence, African patients with syndromic craniosynostosis are mostly diagnosed based on clinical presentations, without confirmatory sequencing of FGFR genes. As a result, African‐specific allele frequencies, penetrance and expressivity estimates, and possible modifier variants for FGFR‐related craniosynostosis remain largely uncharacterized. The lack of African‐specific genomic data leads to the extrapolation of genetic counseling, prenatal diagnosis, and prediction of phenotypic severity for African families from non‐African cohorts. This approach may be unreliable even within genetically similar populations, as phenotypic variability data discussed earlier suggest. Notwithstanding these challenges, African cohorts are highly valuable for deciphering the molecular etiology of FGFR‐related craniosynostosis. In a Congolese family with an affected mother–son pair exhibiting variable expressivity of Apert syndrome, a novel heterozygous triple‐nucleotide substitution in *FGFR2* (c.756_758delGCCinsCTT) was observed [18]. A case report from Nigeria documented the p.Ser252Trp *FGFR2* variant in a patient with Apert syndrome [19].

The current study is aimed at deciphering the genetic etiology of syndromic craniosynostosis in a sub‐Saharan African (Ghanaian) cohort presenting with phenotypes that were characteristic of *FGFR2*‐related syndromes, employing whole exome sequencing (WES). As a hypothesis, we further sought to identify additional genetic risk variants that may contribute to phenotypic variability observed in individuals with FGFR*-*related syndromes. Importantly, these modifier variants had to be linked to inconsistently reported phenotypes observed in individuals with FGFR‐related syndromes. Thus, we opted for WES rather than targeted FGFR sequencing to identify potential contributors to phenotypic variability, such as co‐occurring craniofacial syndromes, that may account for the variable phenotypes observed in our probands. Additionally, we elucidated the structural effects of identified *FGFR2* variants on proteins using HOPE (Have yOur Protein Explained) analysis. Further in silico functional evaluation ascertained the possible functional roles of the implicated genes and variants. The study confirmed the causative roles of previously reported variants in *FGFR2* in the etiology of syndromic craniosynostosis in our cohort. This is a critical, knowledge‐gap‐filling genomics study on *FGFR2*‐related syndromic craniosynostosis in the Ghanaian population and sub‐Saharan Africa more broadly.

## 2. Materials and Methods

### 2.1. Ethical Compliance, Informed Consent, and Study Population

This cross‐sectional observational study employed a family‐based approach, aiming to utilize case–parent trios, which enables detection of de novo variants. Institutional Review Board (IRB) approval for the study was obtained from the Komfo Anokye Teaching Hospital (KATH)‐IRB, Kumasi, Ghana (KATH‐IRB/AP/032/20). Before sample and data collection, participants granted written informed consent. As all probands were minors (below 18 years of age), consent forms were signed by a parent or legal guardian. Each participant was assigned a unique identification (ID) number to ensure confidentiality. A total of eight Ghanaian families were recruited from the Cleft‐Craniofacial Clinic at KATH in Kumasi, Ghana. Eligible probands were those who presented with the clinical presentation of FGFR*-*related craniosynostosis at the clinic during the study period, from September 2020 to June 2023. Participants were recruited using a convenience sampling approach, with recruitment conducted consecutively based on their availability, given the rarity of the condition. As the study utilized a case–parent trio approach, DNA samples were obtained from probands and parents. However, for three families (Families 4, 7, and 8), fathers were unavailable to participate, limiting the detection of de novo variants in these families.

Systematic phenotyping of probands was carried out by a multidisciplinary Cleft‐Craniofacial Team at the National Cleft Care Center (NCCC) at KATH, Kumasi, Ghana. The team comprises a craniofacial surgeon, oral and maxillofacial surgeons, pediatricians, a geneticist, ENT (ear, nose, and throat) specialists, speech therapists, orthodontists, and other clinical specialists. The diagnosis of craniosynostosis was based on 3D computed tomography (3D‐CT) images of the head or cranium (bone‐only protocol) of the probands. Thus, 3D‐CT images were used to assess the patency or synostosis of each of the sutures of the skull in order to make a diagnosis. Moreover, using established clinical protocols at the NCCC, probands were further assessed for other clinical presentations, including additional craniofacial and extracranial anomalies. These included airway, hearing, genitalia, and limb assessments, intracranial pressure, and, where applicable based on age at recruitment, neurodevelopmental milestones (motor skills, language and communication, cognitive development, and social and emotional development). All reported phenotypes in the current study are presurgical clinical observations. In summary, the phenotypes reported for each proband were based on thorough, systematic clinical evaluation by a multidisciplinary Cleft‐Craniofacial Team, based on the age of the probands at the time of recruitment for the study. Because we could not recontact some patients after the initial recruitment, we could not assess other phenotypes that may have become more apparent later in life.

### 2.2. Sample and Data Collection, DNA Extraction and Quantification, and XY Genotyping

Samples (saliva and cheek swab) were collected from participating individuals utilizing Oragene saliva toolkits (http://www.dnagenotek.com; DNA Genotek, Ottawa, Ontario, Canada). Samples were collected from probands and their parents. However, for three families (Families 4, 7, and 8), we could not obtain samples from fathers because they were unavailable. Clinical, family history, and other information were obtained using a questionnaire.

The protocols for DNA extraction and quantification and XY genotyping have been previously published by our group [20]. To summarize, DNA was extracted from saliva/cheek swab samples utilizing the Oragene DNA extraction protocol, which sequentially encompassed overnight incubation of samples, cell lysis and purification of DNA from lysate, ethanol precipitation of DNA, and resuspension of DNA in EDTA elution buffer. Qubit 4 Fluorometer and Qubit assay kits (Thermo Fisher Scientific, Waltham, Massachusetts, United States) were used to quantify DNA samples. As a quality control measure, the sex of study participants was verified using XY genotyping through PCR amplification of specific loci on the Y chromosome (*USP9Y*) and X chromosome (*KDM6A*) [20].

### 2.3. WES of DNA Samples

WES was carried out using 2 *μ*g DNA samples at Azenta Life Sciences (Azenta, United States), as previously described [21]. Following the manufacturer′s instructions, library preparation was performed using the Twist Human Comprehensive Exome kit (Twist Bioscience, South San Francisco, California, United States). The Covaris S220 system was utilized to acoustically fragment genomic DNA samples, followed by purification and end‐repair of DNA fragments. A‐tailing of fragments facilitated their ligation with the T‐overhang of sequencing adapters. An Agilent TapeStation (Agilent Technologies, Palo Alto, California, United States) was used to validate adapter‐ligated DNA, followed by quantification with a Qubit 4 Fluorometer.

Target enrichment was accomplished by hybridizing adapter‐ligated fragments with biotinylated baits, followed by streptavidin‐coated bead capture. Captured DNA was washed, PCR‐amplified, and indexed with Illumina primers. Quality control checks on postcapture libraries utilized Agilent TapeStation, and quantification was performed with Qubit 4 Fluorometer and real‐time PCR (Kapa Biosystems, Wilmington, Massachusetts, United States). Subsequently, multiplexed libraries were clustered onto flow cell lanes and sequenced on an Illumina HiSeq platform using a 2 × 150 bp paired‐end configuration at 100x coverage, according to the manufacturer′s instructions. The HiSeq Control Software (HCS) was utilized for image analysis and base calling. Illumina′s bcl2fastq V2.19 software was used to convert raw sequence data (.bcl files) to FASTQ format, followed by demultiplexing, permitting one mismatch for index identification.

### 2.4. Bioinformatics Analyses: Variant Calling

FastQC V0.11.9 [22] was utilized for quality control checks on the raw sequence reads. Trimmomatic V0.39 [23] was used to trim low‐quality reads and adapter sequences. BWA‐MEM of Sentieon V202112.01 [24] was utilized to align reads that passed a quality threshold (*Q* > 30) to the human reference genome GRCh38. Sentieon′s Sort utility and Dedup tools were utilized to sort the resulting alignments and mark PCR duplicates, respectively. Sentieon DNAscope algorithm (V202112.01) was employed for variant calling, including insertions/deletions (indels) and single‐nucleotide variants (SNVs). BCFtools V1.13 [25] was used for the subsequent normalization of generated VCF files, left‐aligning of indels, and splitting of multiallelic sites into separate entries. Lastly, for each variant, Ensembl VEP V104 [26] was used to identify overlapping transcripts and to predict the functional impact of the variants on those transcripts.

### 2.5. Variant Classification and Prioritization

Variants were classified and prioritized utilizing the American College of Medical Genetics and Genomics (ACMG)/Association of Molecular Pathology (AMP) guidelines [27] (Figure 1). The 1000 Genomes Project (http://www.internationalgenomes.org), Exome Sequencing Project (ESP; https://bio.tools/esp), African Genome Variation Database (https://agvd.h3abionet.org), and Genome Aggregation Database (gnomAD) V4.1 (http://gnomad.broadinstitute.org) were used to first filter for rare variants, defined as those with a minor allele frequency (MAF) below 1%, consistent with the rarity of craniosynostosis. Prior to downstream analysis, we filtered for missense, frameshift, splice‐site, start‐gain/loss, and stop‐gain/loss variants.

**Figure 1:**
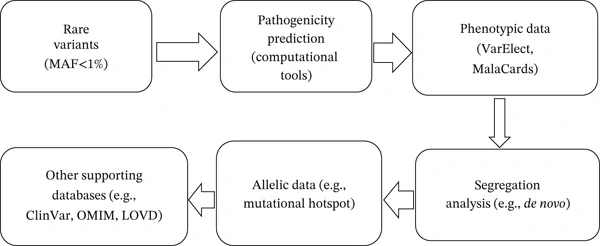
Variant classification and prioritization workflow.

To identify likely pathogenic variants, we applied computational prediction tools using defined thresholds (Supporting Information 5: Table S1). The pathogenicity of missense variants was evaluated with 11 in silico predictors (SIFT V5.2.2, PolyPhen‐2 V2, CADD V1.4, ClinPred, MetaRNN, REVEL, BayesDel_addAF, AlphaMissense, MutPred2, MutationAssessor, and MutationTaster rankscore) available via the dbNSFP V4.9 database [28]. The choice of these in silico tools was based on previous sensitivity and specificity analyses [29–31] of their performance on craniofacially important variants. Variants predicted to be likely pathogenic by at least six of these tools were investigated further. The requirement of at least six tools to predict pathogenicity for classifying a variant as pathogenic was based on our in‐house pipeline [21, 32], which was informed by the varied performance of these tools due to their underlying algorithms [29–31]. SpliceAI V1.3.1 [33] was employed to assess the pathogenicity of splice‐site variants. Additionally, CADD was used to predict the functional impact of stop‐gain/loss and start‐gain/loss variants.

Furthermore, we deciphered the biological significance and functional evidence of genes harboring implicated likely variants utilizing various databases, including VarElect NGS Phenotyper V5.26 (https://ve.genecards.org/), GeneCards V5.26.0 (https://www.genecards.org/) [34], MalaCards V5.26 (https://www.malacards.org/), Mouse Genome Informatics (MGI; https://www.informatics.jax.org), Alliance of Genome Resources (https://www.alliancegenome.org), DECIPHER V11.38 (https://www.deciphergenomics.org), Online Mendelian Inheritance in Man (OMIM; https://www.omim.org), and FaceBase (https://www.facebase.org/) [35]. These databases gave further evidence of human diseases linked to implicated genes, as well as experimental observations in animal models. For example, VarElect utilizes various sources of information to decipher the role of genes in diseases/phenotypes, contingent upon protein interaction networks, shared biological pathways, paralogous relationships, and scientific literature.

Lastly, segregation analysis was performed using family pedigrees and WES genotype data. In this craniosynostosis cohort, *de novo* variants were prioritized due to a lack of family history of craniosynostosis in the family. To further confirm the pathogenicity of the implicated variants, they were assessed using available allele‐level data to determine whether they occurred in mutational hotspots or had been previously classified as likely pathogenic or pathogenic for certain diseases. This is aimed at strengthening the clinical significance of the prioritized variants. Databases used for this filtering comprised ClinVar (https://www.ncbi.nlm.nih.gov/clinvar/) and Global Variome shared LOVD (https://www.lovd.nl).

As a quality control measure, the p.Gly272del (c.815_817del) variant observed in *FGFR2* was validated using our previously published Sanger sequencing protocol [36].

### 2.6. Protein Structure Analysis of FGFR2

To determine the structural and functional impact of the identified variants on FGFR2 protein, an in silico tool, HOPE [37], was employed. During HOPE prediction, the FGFR2 protein sequence in FASTA format or the accession code retrieved from UniProt (https://www.uniprot.org) was uploaded to the web interface. The mutant amino acid position was selected, with the wildtype amino acid changed to the mutant amino acid, followed by a query submission for analysis.

### 2.7. Spatiotemporal Gene Expression Analysis for Implicated Genes

To derive functional insights, gene expression analysis for *FGFR2* and possible phenotypic modifier genes was performed using the Gene Expression Database (GXD; https://www.informatics.jax.org/expression.shtml) embedded in the MGI database (https://www.informatics.jax.org). Both *FGFR2* and the hypothetical modifier genes were included in the analyses to ascertain how the expression of the plausible modifier genes compares with that of *FGFR2*. The “Batch query” was executed by uploading candidate genes. The chosen structures included cranial sutures (e.g., coronal and sagittal), calvarial bones, skull base, cranial mesenchyme, and first and second branchial arches from which the maxillary and mandibular prominences are derived. The branchial arches were included because they give rise to craniofacial skeletal elements. These bones contribute directly to the skull vault and midface. Dysregulation in these regions underlies facial dysmorphism commonly seen in craniosynostosis [8].

Theiler stages TS17–TS26 (approximately E10.5–E18.0) were queried to capture dynamic gene expression across embryonic skull and facial morphogenesis, from neural crest migration and branchial arch patterning (TS17–TS20) through suture initiation, ossification, and facial fusion (TS21–TS26). This temporal window is essential because branchial arch fusion and cranial suture patency are time‐sensitive processes, and misexpression at these stages can lead to premature fusion or midline defects observed in patients [8].

To corroborate observations in the mouse model, a human craniofacial atlas dataset (https://cotneyshiny.research.chop.edu/shiny-apps/craniofacial_all_snRNA_v2/; assessed on April 6, 2026) was used to ascertain the spatiotemporal expression of the genes. This single‐nucleus RNA (snRNA)‐Seq data demonstrates gene expression patterns across several craniofacial cell lineages, as well as developmental time points, particularly Carnegie Stages (CS) 12–20. CS 12–20 correspond to 4–8 weeks of embryonic development, when most craniofacial structures form. The Shiny2 tool, an R package embedded in the website, was utilized to generate the heatmap. The rationale for this human craniofacial expression analysis was to demonstrate the developmental relevance of potential modifier genes and *FGFR2* to craniofacial development.

### 2.8. Pathway Enrichment and Network Analyses

The pathway analysis basically subjected the prioritized genes to functional enrichment analysis using g:Profiler [38], a tool that identifies statistically overrepresented biological pathways. Also, Gene Ontology (GO) terms are identified by cross‐referencing gene sets against curated databases, including KEGG, Reactome, and WikiPathways. Significantly enriched terms that have adjusted *p* value < 0.05 and corrected for multiple testing via the g:SCS threshold method were generated, visualized, and interpreted. The resulting enrichment GEM file was subsequently imported into Cytoscape V3.10.3 [39] to generate a pathway interaction network using the EnrichmentMap plugin (V3.5). EnrichmentMap clusters overlapping pathways into functional themes, with edges representing similarity between terms. This enables the identification of core biological processes or signaling cascades dysregulated in syndromic craniosynostosis.

### 2.9. Protein–Protein Interaction (PPI) Network and Hub Gene Identification

In exploring functional interactions among prioritized genes, a PPI network was constructed using the STRING database [40], which integrates experimentally validated interactions, coexpression data, and literature‐derived associations. The network was generated using a high‐confidence interaction‐score threshold to minimize noise. The resulting PPI network was imported into Cytoscape for topological analysis and refinement. cytoHubba (V0.1), a plugin for identifying critical nodes, was used to rank hub genes using the Maximal Clique Centrality (MCC) algorithm. Hub genes, those with the highest centrality scores, were inferred as potential regulators or key drivers of the network, given their extensive connectivity and pivotal roles in maintaining network integrity.

## 3. Results

### 3.1. Clinical Presentations of Study Participants

A multidisciplinary Cleft‐Craniofacial Team performed clinical assessments as described in the Methods. All eight recruited families were simplex ones with no reported family history of craniosynostosis, whether syndromic or nonsyndromic (Supporting Information 1: Figure S1). Clinically, the probands of four of the families were diagnosed with Crouzon syndrome (Families 1, 2, 4, and 5), whereas the other four were diagnosed with Apert syndrome (Families 3, 6, 7, and 8). At the time of participant recruitment, the clinical diagnosis was based solely on clinical presentations; no clinical genetic testing had been performed, as such tests are lacking in the Ghanaian healthcare system. Craniosynostosis affecting the coronal suture was observed in all probands. However, there was significant phenotype variability even in probands diagnosed with the same syndrome (Table 1). Importantly, the age of probands at the time of recruitment may have influenced the observed phenotypes. For example, ~63% (5/8) of probands were recruited before Age 3, at which time many phenotypes may not be well developed to be clinically obvious. Such phenotypes include (a) malocclusion, which could not be assessed in these 63% of probands because the primary dentition develops at ~0.5–3 years, and (b) neurodevelopmental disorders, which may become more obvious by Ages 3–5. Accordingly, phenotypes listed as absent in Table 1 should be interpreted with caution, as this may reflect their absence at the time of clinical evaluation rather than their absence throughout the proband′s lifespan.

**Table 1:** Summary of the clinical presentations of the probands.

Clinical presentations	Probands of families							
	F1	F2	F3	F4	F5	F6	F7	F8
Craniosynostosis (affected sutures)								
Metopic suture	−	+	−	−	+	+	−	+
Coronal suture	+	+	+	+	+	+	+	+
Sagittal suture	+	+	+	−	+	−	−	+
Lambdoid suture	−	+	−	−	−	−	−	+
Other craniofacial and dental dysmorphisms								
Cleft palate	−	−	−	−	−	−	−	+
High‐arched palate	−	+	+	−	−	+	+	+
Proptosis/exophthalmos	+	+	−	+	+	+	−	+
Midface retrusion/deficiency/hypoplasia	+	+	−	+	−	−	−	+
Malocclusion	+	−	−	−	−	−	−	+
Flattened zygomatic arch	+	−	−	−	−	−	−	−
Retruded forehead	+	−	−	−	−	−	−	−
Protruded mandible/mandibular prognathism	+	−	−	−	−	−	−	+
Bulging anterior fontanelle	−	+	−	−	−	−	−	−
Low‐set ears	−	−	−	−	−	+	−	+
Frontal bossing	−	−	−	−	−	−	−	+
Flat posterior skull	−	−	−	−	−	−	−	+
Beaked nose	−	−	−	−	−	−	−	+
Downward‐slanting palpebral fissure	−	−	−	−	−	−	−	+
Ocular hypertelorism	−	−	−	−	−	−	−	+
Patchy eyebrows	−	−	−	−	−	−	−	+
Limb abnormalities								
Finger syndactyly	−	−	+	−	−	+	+	+
Toe syndactyly	−	−	+	−	−	+	+	+
Symphalangism	−	−	+	−	−	+	+	+
Auditory and visual anomalies								
Hearing loss	−	−	−	−	−	−	−	−
Visual impairment	−	−	−	−	−	−	−	−
Neurodevelopmental disorders								
Developmental delay	−	−	−	−	−	−	−	−
Intellectual disability	−	−	−	−	−	−	−	+
Other abnormalities observed								
Intracranial hypertension	−	−	−	−	−	−	−	−
Airway involvement (difficulty breathing)	−	+	−	−	−	−	−	−
Sleep apnea	−	+	−	−	−	−	−	−

*Note:* The symbol “+” indicates clinical presentation present in proband; the symbol “−” indicates clinical presentation absent in proband. Probands with Crouzon syndrome: F1, F2, F4, and F5. Probands with Apert syndrome: F3, F6, F7, and F8. All coronal suture synostoses were bilateral, except for proband F7, where it occurred as a left unilateral coronal synostosis. The probands with lambdoid suture synostosis were bilateral in presentation. Probands F2, F3, F4, F6, and F7 were recruited before Age 3, at which time some clinical phenotypes may not be obvious. For this reason, the indicated absence of a phenotype must be interpreted with caution. This is because those phenotypes may manifest later in life. For example, malocclusion may become obvious at Age 3, when the primary dentition fully forms.

Abbreviation: F, family.

*Family 1*: The female proband was recruited at Age 12. She presented with craniosynostosis affecting the bilateral coronal and sagittal sutures. Other observed phenotypes included malocclusion, flattened zygomatic arch, retruded forehead, midface deficiency, protruded mandible, and exophthalmos or proptosis.

*Family 2*: This male proband, recruited at 1 year and 8 months of age, presented with craniosynostosis that impacted all the cranial sutures based on a 3D‐CT scan. Proptosis, bulging anterior fontanelle, retarded midfacial growth, sleep apnea, difficulty breathing, and high‐arched palate were the other clinical phenotypes in this proband.

*Family 3*: Recruited at ~2 months of age, the male proband presented with craniosynostosis affecting the bilateral coronal and sagittal sutures. Other clinical presentations included syndactyly of fingers and toes, symphalangism, and a high‐arched palate.

*Family 4*: The male proband was recruited at ~1 month, presenting with bilateral coronal craniosynostosis, as well as proptosis and midface hypoplasia.

*Family 5*: The proband presented with craniosynostosis affecting the sagittal, bilateral coronal, and metopic sutures, as well as proptosis. She was recruited at the age of 3 years.

*Family 6*: The female proband was recruited at 1 year and 6 months of age. She presented with craniosynostosis involving the bilateral coronal and metopic sutures. Other observed phenotypes were proptosis, high‐arched palate, syndactyly of fingers and toes, symphalangism, and low‐set ears.

*Family 7*: Left coronal craniosynostosis, syndactyly of fingers and toes, symphalangism, and a V‐shaped, high‐arched palate were the clinical presentations observed in the female proband recruited at 1 year and 10 months of age.

*Family 8*: The male proband was recruited at Age 3. The observed craniosynostosis affected all cranial sutures, as seen on a 3D CT head (bone‐only) scan. Other observed phenotypes include complete cleft palate, frontal bossing, flat posterior skull, retarded midfacial growth, wide, low‐set ears, beaked nose, proptosis, downward‐slanting palpebral fissure, ocular hypertelorism, patches of missing hair of eyebrows, mandibular prognathism (maxilla about 1 cm shorter than mandible), crowded teeth (malocclusion), high‐arched palate, syndactyly of toes and fingers, and symphalangism, as well as mild intellectual disability.

### 3.2. Etiologic Risk Variants Observed in *FGFR2*

WES was carried out at an average read depth of 100x using a paired‐end configuration. Quality control checks on the WES dataset demonstrated highly accurate reads with the following average metrics: (a) > 95% of bases with base quality score distributions of *Q* > 35, (b) over 95% of reads having quality scores of *Q* > 35, and (c) ~97% of reads mapping to their target regions. After variant calling, an average of 33,000 SNVs and indels were observed per sample. These variants were filtered to obtain the probable etiologic variants.

The variant filtering procedure, based on the ACMG/AMP guidelines, included variant annotation, classification, and prioritization. De novo variants were prioritized because the cohort lacked a family history of craniosynostosis. Moreover, inherited variants were also evaluated, considering both variable penetrance and expressivity. This procedure identified pathogenic variants in *FGFR2* in all eight probands (Table 2). These variants have MAF < 1%, are predicted to be pathogenic by at least six tools, and are linked to craniosynostosis and other craniofacial phenotypes (Supporting Information 6: Table S2, Supporting Information 7: Table S3, and Supporting Information 8: Table S4). In five families (Families 1, 2, 3, 5, and 6) in which both paternal and maternal samples were available, the observed *FGFR2* variants were de novo, consistent with the existing literature. However, in three families (Families 4, 7, and 8), paternal samples were unavailable, limiting our ability to confirm whether these variants are de novo. Whereas most of the observed variants (p.Asp336Gly, p.Cys342Ser, p.Pro253Arg, p.Cys342Gly, and p.Ser252Trp) were missense variants, one variant (p.Gly272del) was an in‐frame deletion. All six observed variants have been previously implicated in the etiology of *FGFR2*‐related syndromes, particularly Apert and Crouzon syndromes. VarSome (https://varsome.com) predicts the p.Gly272del variant to be likely pathogenic (Supporting Information 2: Figure S2). It was also confirmed by Sanger sequencing in both forward and reverse directions (Supporting Information 3: Figure S3).

**Table 2:** Spectrum of *FGFR2* variants associated with syndromic craniosynostosis.

Family ID	Genomic coordinate (rs number)	HGVSc	HGVSp	NTP
Family 1 (GH20209003)	Chr10:121517396 (rs1057519042)	c.1007A>G	p.Asp336Gly	11
Family 2 (GH20209005)	Chr10:121517378 (rs121918487)	c.1025G>C	p.Cys342Ser	11
Family 3 (GH20209010)	Chr10:121520160 (rs77543610)	c.758C>G	p.Pro253Arg	9
Family 4 (GH20209011)	Chr10:121517379^a^ (rs121918488)	c.1024T>G	p.Cys342Gly	11
Family 5 (GH20209015)	Chr10:121520100	c.815_817delGAG	p.Gly272del	N/A
Family 6 (GH20229016)	Chr10:121520163 (rs79184941)	c.755C>G	p.Ser252Trp	11
Family 7 (GH20229017)	Chr10:121520163^a^ (rs79184941)	c.755C>G	p.Ser252Trp	11
Family 8 (GH20135053)	Chr10:121520163^a^ (rs79184941)	c.755C>G	p.Ser252Trp	11

*Note:* All variants were observed in the heterozygous state. All analyses were based on Ensembl Transcript ID ENST00000358487.

Abbreviations: N/A, not applicable; NTP, number of tools predicting pathogenicity.

^a^Variant not in mother, but paternal samples were not available to determine if the variant was de novo or not.

### 3.3. Distribution of *FGFR2* Variants by Syndromes, Cranial Sutures, and Protein Domains

The frequency and distribution of *FGFR2* variants varied across craniosynostosis syndromes and cranial suture types (Figure 2). Patients with both Apert and Crouzon syndromes had a total variant frequency of 4 (Figure 2A), with Apert syndrome cases predominantly (75%, *n* = 3) harboring the p.Ser252Trp variant, consistent with existing literature. However, Crouzon syndrome cases exhibited greater allelic diversity, with four distinct variants each occurring at equal frequency. Among the cranial suture types, the coronal suture was affected in all probands (100%, *n* = 8), with all six different variants linked to premature fusion of this suture (Figure 2B). The lambdoid suture was the least affected suture type (25%, *n* = 2) in this cohort, being negatively impacted by two pathogenic variants (p.Ser252Trp and p.Cys342Ser). Notably, p.Ser252Trp emerged as the most frequently observed variant across both syndrome types and cranial suture types, suggesting its potential significance in the molecular pathogenesis of craniosynostosis. However, ascertainment bias may contribute to this observation, as children with more striking phenotypes are easier to identify. Interestingly, all the observed variants occurred very close to or within the extracellular Ig‐like C2‐type 3 domain of *FGFR2* (Figure 2C). This domain directly interacts with fibroblast growth factors, and its alternative splicing is crucial for ligand selectivity.

**Figure 2:**
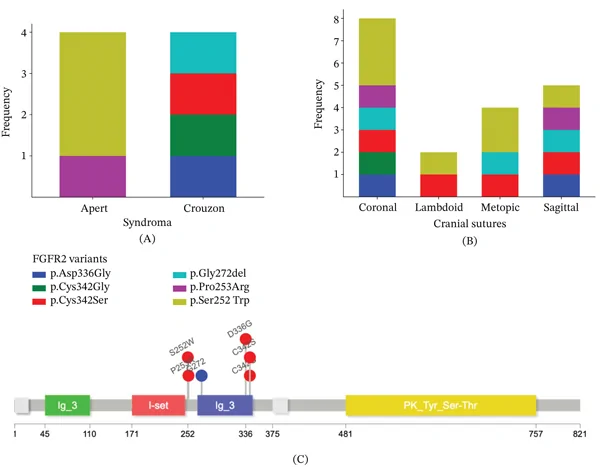
Distribution of *FGFR2* variants by syndrome type, cranial suture involvement, and protein domains. (A) Stacked bar chart showing the frequency of six *FGFR2* variants in patients with Apert and Crouzon syndromes. (B) Stacked bar chart showing the frequency of six *FGFR2* variants in affected cranial suture types. The size of the color represents the frequency of each variant within the respective category. (C) Domain architecture of FGFR2 showing locations of identified variants. Light gray, signal peptide region; deep gray regions, transmembrane regions; green, Ig_3 (immunoglobulin‐like Domain 3); blue, immunoglobulin domain; red, immunoglobulin I‐set (linker) domain; yellow, protein tyrosine and serine/threonine kinase domain.

### 3.4. Probable Genetic Modifiers Contributing to Phenotypic Variability

Phenotypes reported in patients with Apert and Crouzon syndromes, both in our study and previously, may be variable. We hypothesized that additional etiologic variants may account for some craniofacial, neurodevelopmental, hearing, and other phenotypes that have been observed inconsistently with craniosynostosis in such patients. Therefore, we searched our WES dataset for additional pathogenic variants, beyond those in *FGFR2*, that may explain phenotypic variability. These variants could explain variable phenotypes by causing co‐occurring syndromes or acting as phenotypic modifiers. This will be especially true for phenotypes that are rarely or inconsistently reported for Apert and Crouzon syndromes. Based on this, we identified additional pathogenic variants in other genes (Table 3), whose contribution to the etiology of craniosynostosis and associated phenotypes cannot be discounted (Supporting Information 7: Table S3 and Supporting Information 8: Table S4). Nevertheless, *FGFR2* was prioritized as the primary driver of craniosynostosis, though the other genes in Table 3 may account for variability in clinical phenotypes.

**Table 3:** Candidate variants potentially contributing to phenotypic variability.

Family ID	Genomic coordinate	Gene	Genotype	Transcript ID	HGVSc	HGVSp	NTP
Family 1 (GH20209003)	Chr22:19973288^d^ (rs758305598)	*ARVCF*	Het.	ENST00000263207	c.2269A>C	p.Asn757His	7
	Chr1:1645163^d^ (novel)	*CDK11B*	Hom.	ENST00000341832	c.593_594insGGAGG	p.Arg202SerfsTer19	N/A
	Chr3:184971748^a^ (rs758939120)	*VPS8*	Hom.	ENST00000625842	c.3416G>A	p.Arg1139His	7

Family 2 (GH20209005)	Chr16:84873019^b^ (novel)	*CRISPLD2*	Het.	ENST00000262424	c.1009C>A	p.His337Asn	11

Family 3 (GH20209010)	Chr14:104945000^d^ (rs779995601)	*AHNAK2*	Het.	ENST00000333244	c.10450_10451insTACA	p.Gly3484ValfsTer38	N/A
	Chr15:41866454^b^ (rs974936065)	*SPTBN5* ^e^	Het.	ENST00000320955	c.6520G>T	p.Glu2174Ter	N/A
	Chr15:41879329^c^ (novel)		Het.		c.3113T>C	p.Leu1038Pro	9

Family 4^f^ (GH20209011)	Chr1:21824556 (novel)	*HSPG2*	Het.	ENST00000374695	c.12725T>G	p.Leu4242Arg	11
	Chr13:20189488^b^ (rs371024165)	*GJB2*	Het.	ENST00000382848	c.94C>T	p.Arg32Cys	11
	Chr17:82941446 (novel)	*TBCD*	Het.	ENST00000355528	c.3527A>C	p.Asp1176Ala	7
	Chr22:46974368 (rs1333784593)	*TBC1D22A*	Het.	ENST00000337137	c.1094G>C	p.Trp365Ser	11
	Chr6:109162950 (novel)	*CEP57L1*	Het.	ENST00000517392	c.1366_1367dup	p.Met457SerfsTer14	N/A
	Chr14:104951131 (rs531942140)	*AHNAK2*	Het.	ENST00000333244	c.4319_4320insG	p.Asp1442ArgfsTer10	N/A

Family 5 (GH20209015)	Chr7:102483656^d^ (rs1554581975)	*RASA4B*	Het.	ENST00000465829	c.2348A>G	p.His783Arg	6
	Chr14:104948679^d^ (novel)	*AHNAK2* ^e^	Het.	ENST00000333244	c.6771_6772insGG	p.Ile2258GlyfsTer9	N/A
	Chr14:104948682^d^ (rs1215492197)		Hom.		c.6765_6768del	p.Ile2256ThrfsTer9	N/A
	Chr11:111875482^c^ (rs1242596116)	*FDXACB1* ^e^	Het.	ENST00000260257	c.1314del	p.Lys438AsnfsTer12	N/A
	Chr11:111875813^b^ (novel)		Het.		c.983del	p.Gly328GlufsTer25	N/A

Family 6 (GH20229016)	Chr8:93772596^d^ (novel)	*TMEM67*	Het.	ENST00000453321	c.659C>A	p.Ser220Tyr	8

Family 7^f^ (GH20229017)	Chr19:12661286 (novel)	*MAN2B1*	Het.	ENST00000456935	c.1000A>G	p.Lys334Glu	9

Family 8^f^ (GH20135053)	Chr1:149464092 (rs1306186424)	*NOTCH2NLC*	Het.	ENST00000650865	c.823del	p.Thr275GlnfsTer29	N/A

Abbreviations: Hem., hemizygous; Het., heterozygous; Hom., homozygous; N/A, not applicable; NTP, number of tools predicting pathogenicity.

^a^Both parents are heterozygous for the variant.

^b^Inherited from mother.

^c^Inherited from father.

^d^De novo variant.

^e^Patient is compound heterozygous for the variants.

^f^Paternal samples were not available to determine if the variant was de novo or not.

The variants reported in Table 3 must be interpreted with caution, given their associated disease conditions and modes of inheritance, although not all have been linked to Mendelian disorders in OMIM. For example, OMIM associates HSPG2 variants with autosomal recessive conditions (Supporting Information 8: Table S4). For this reason, the heterozygous carrier in Family 4 is not likely to show the expected syndrome. This also applies to variants in genes such as *TBCD*, *TMEM67*, and *MAN2B1*, which are associated with autosomal recessive conditions, but the current probands harbored heterozygous variants. In OMIM, variants in *GJB2* cause mostly autosomal dominant conditions, although some are linked to autosomal recessive conditions (Supporting Information 8: Table S4). In ClinVar, the reported *GJB2* variant in Table 3 (p.Arg32Cys; ClinVar ID: 188758) has been linked to both autosomal recessive nonsyndromic hearing loss 1A and autosomal dominant keratitis–ichthyosis–hearing loss syndrome/autosomal dominant nonsyndromic hearing loss 3A. However, most reported ClinVar studies found the variant in a homozygous or compound heterozygous state, suggesting a predominance of an autosomal recessive inheritance pattern for this variant. Nevertheless, OMIM information, including the inheritance pattern, may change as more evidence emerges. For other genes (*ARVCF*, *CDK11B*, *VPS8*, *CRISPLD2*, *AHNAK2*, *SPTBN5*, *TBC1D22A*, *CEP57L1*, *RASA4B*, and *FDXACB1*), variants in them have not previously been linked to any syndrome or disease in OMIM or ClinVar, and their mode of inheritance is unknown. However, these genes have been associated with interesting craniofacial and other phenotypes in databases such as DECIPHER, MGI, and AGR (Supporting Information 7: Table S3 and Supporting Information 8: Table S4). Thus, functional characterization is required to decipher genotype–phenotype correlations for these genes. Again, genes with frameshift variants had varied constraint metrics (specifically pLI scores) in gnomAD: *CDK11B* (0.02), *AHNAK2* (0), *CEP57L1* (0), *FDXACB1* (0), and *NOTCH2NLC* (0.96). This largely suggests that, apart from the frameshift variants in *NOTCH2NLC* (whose pLI score of 0.96 suggests it is intolerant to heterozygous frameshift variants) and *FDXACB1* (which has compound heterozygous frameshift variants), all other heterozygous frameshift variants should be interpreted with caution because of limited constraints. However, pLI score alone is not a sufficient metric in determining variant pathogenicity, and this metric must be interpreted together with the ACMG codes and associated phenotypes in Supporting Information 7: Table S3 and Supporting Information 8: Table S4. The compound heterozygous variants in *FDXACB1* and *SPTBN5* were inherited from either parent, making them phase‐aware. Lastly, variants reported for Families 4, 7, and 8 must be interpreted with caution, as no paternal sample was available to support full segregation analysis, though these variants were absent in the mother. In summary, although the variants reported in Table 3 may contribute to phenotypic variability in the probands, they should be interpreted with caution based on the inheritance patterns of associated diseases and constraint metrics for frameshift variants.

### 3.5. Enriched Pathways, Enrichment Map, Interactome, and Hub Genes Associated With Syndromic Craniosynostosis

Pathway enrichment analysis revealed functionally enriched HPO terms, but no enrichment for GO or other terms was observed for genes implicated in the study (Figure 3). The enriched HPO terms were expected, as the genes harboring variants of interest were selected from the WES dataset based on the phenotypes of the probands. Beyond HPO categories, this analysis is usually useful for identifying enriched pathways, such as GO: biological and GO: molecular. However, no enriched biological process (GO: BP) or molecular function (GO: MF) was observed, although HPO categories were enriched. Many enriched human phenotypes (HPs) were observed, including “conductive hearing impairment,” “functional abnormality of the middle ear,” “abnormal cerebral ventricle morphology,” and “corneal neovascularization.” Key genes that drive this phenotypic enrichment include *ARVCF*, *FGFR2*, *GJB2*, *HSPG2*, *MAN2B1*, *NOTCH2NLC*, *TBCD*, and *TMEM67* (Supporting Information 4: Figure S4). The enrichment map (Figure 3B) illustrates the functional relationships among the HPO categories. Together, these visualizations provide insight into the phenotypic consequences of the genetic variants identified in the current study.

**Figure 3:**
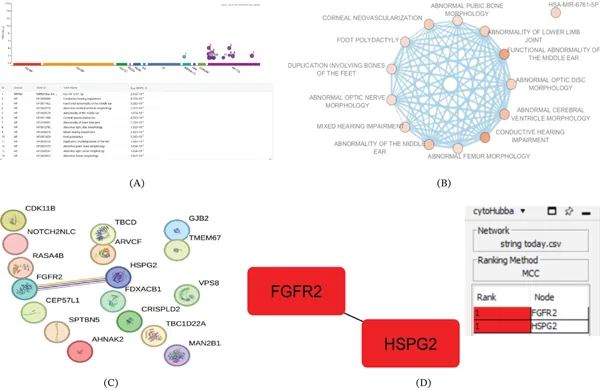
Pathway enrichment analysis, interactome, and hub genes. (A) Significantly enriched Gene Ontology (GO) terms and phenotypes associated with implicated genes retrieved from g:Profiler. The *y*‐axis denotes −log10 adjusted *p* values, whereas the *x*‐axis indicates distinct functional categories. Dot size corresponds to the number of genes within each term, and numbered dots identify key enriched terms detailed in the accompanying table. (B) Pathway enrichment map by Cytoscape illustrating functional relationships among significantly enriched HPO terms derived from gene set enrichment analysis. Each node denotes a distinct biological pathway, and edges represent shared genes or functional associations between pathways. (C) Protein–protein interaction (PPI) network from STRING. Nodes indicate proteins, and lines/edges indicate interactions between proteins. (D) Hub genes within the PPI generated from Cytoscape. Red color denotes highly connected genes/hub genes.

PPI analysis among the implicated genes identified only one cluster comprising *FGFR2* and *HSPG2* (Figure 3C). This observation suggests the implicated genes may be components of different pathways. However, their individual functions can produce convergent phenotypes. Alternatively, the small number of genes used for the enrichment analysis may have limited the capacity to identify any enriched pathways. Furthermore, hub gene analysis revealed that *FGFR2* and *HSPG2* may play crucial roles in the interactome (Figure 3D), serving as critical regulatory nodes within the network.

### 3.6. Spatiotemporal Expression Patterns of Implicated Genes

In mouse models, *Cdk11b*, *Cep57l1*, *Crispld2*, *Fdxacb1*, *Fgfr2*, *Gjb2*, *Hspg2*, *Man2b1*, *Tbc1d22a*, *Tbcd*, *Tmem67*, and *Vps8* showed expression in the head mesenchyme, as well as the mandibular and maxillary components of the first pharyngeal arches (Figure 4), which are critical for cranium formation. These expression patterns suggest that these genes may influence craniofacial morphogenesis and ultimately craniofacial phenotypes.

**Figure 4:**
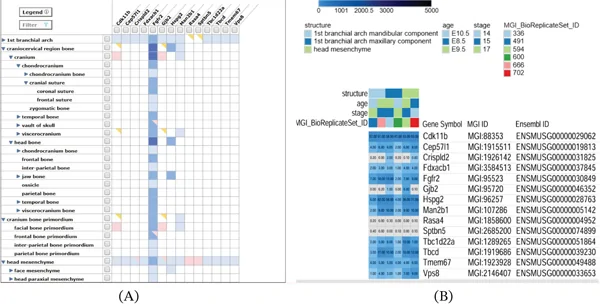
Expression profiles of candidate genes in craniofacial tissues during early mouse embryogenesis. (A) Grid summary of curated expression annotations from MGI GXD for candidate gene set across key craniofacial structures. Blue shades indicate the presence of expression, red cells indicate the absence, and white boxes depict the unavailability of datasets. Shade intensity corresponds to the number of supporting annotations. (B) Heatmap visualization of gene expression levels across early embryonic craniofacial tissues in the mouse, generated using transcriptomic data from the GXD of the MGI platform. *NOTCH2NLC* is not shown here because there is no functional ortholog in mice. Moreover, *Ahnak2* and *Arvcf* are not shown because there is no expression data on the genes as far as the selected tissues are concerned.

In human embryonic craniofacial tissues, the implicated genes are expressed during key developmental stages and in specific cell lineages (Figure 5). Most of the genes (e.g., *GJB2* and *SPTBN5*) are expressed at CS 20, whereas others (e.g., *ARVCF* and *CDK11B*) are active earlier, around the middle of this period (CS 14–16). Furthermore, the expression patterns of these genes differ across various craniofacial cell lineages, such as cranial neural crest cells (CNCCs), osteoblasts, cartilage, auditory, palate, and neural progenitor cells. The spatiotemporal expression patterns indicate they may have distinct roles at different times and in different tissues during human craniofacial development, potentially affecting craniofacial phenotypes.

**Figure 5:**
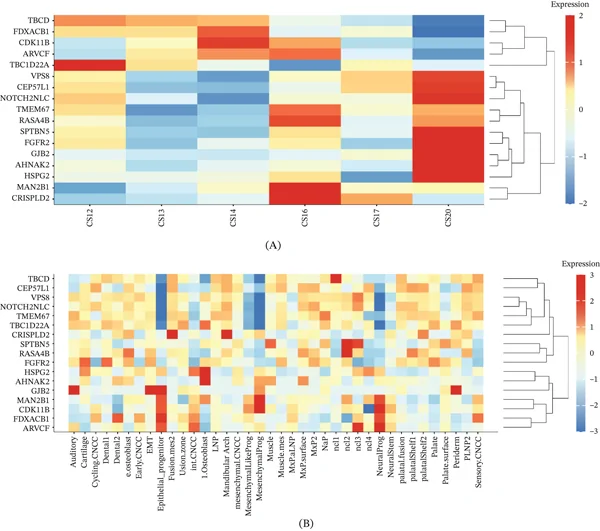
Human craniofacial expression of genes in different cell lineages and time points. A single‐nucleus RNA‐Seq dataset was used. (A) Expression of genes at different developmental stages, corresponding to Carnegie stages (CS) 12–20. CS 12–20 correspond to Weeks 4–8 of human embryonic development, when the palate, lip, and many craniofacial structures form. (B) Expression pattern of genes in various cell lineages from various craniofacial regions. CS: Carnegie stages, cycling. CNCC: cycling cranial neural crest cells, early. CNCC: early cranial neural crest cells, EMT: epithelial‐to‐mesenchymal transition cells, e‐osteoblast: early osteoblast, Fusion.mes2: fusion mesenchymal cells (2), i‐osteoblast: intermediate osteoblast, int. CNCC: intermediate cranial neural crest cells, LNP: lateral nasal process, mesenchymal. CNCC: cranial neural crest cell‐derived mesenchymal cells, MesenchymalLikeProg: mesenchymal‐like progenitor cells, MxP2: maxillary prominence (Cluster 2), MxP.aLNP: maxillary prominence adjacent to lateral nasal process, MxP.surface: maxillary prominence surface cells, NaP: nasal prominence, Nc1/2/3/4: neural crest‐derived cells (Clusters 1, 2, 3, and 4), NeuralProg: neural progenitor cell lines, pLNP: posterior lateral nasal process (Cluster 2), sensory. CNCC: sensory cranial neural crest cells.

### 3.7. In Silico Structural and Functional Impacts of *FGFR2* Variants

The HOPE structural analysis demonstrated how the amino acid substitutions affected FGFR2 stability, interactions, or domain functionality. The modeling considered key features of each variant, including the physicochemical differences between wildtype and mutant amino acid residues, the structural context of the affected site, and the predicted consequences on protein folding, binding interfaces, or domain integrity (Supporting Information 9: Table S5). It was demonstrated that the observed missense variants disrupted normal protein function through alterations in residue size, charge, hydrophobicity, or steric constraints, providing a foundation for prioritizing mutations with the potential pathogenic impact. HOPE could not perform the structural modeling for the p.Gly272del variant because it is not developed for such mutations.

## 4. Discussion

The current study intended to decipher the genetic etiology of syndromic craniosynostosis in a sub‐Saharan African cohort. The eight recruited probands presented with clinical features that were characteristic of *FGFR2*‐related syndromes (Table 1). This ascertainment strategy suggests that our cohort was enriched for the detection of *FGFR2* variants. Therefore, identifying *FGFR2* variants in all eight probands does not necessarily demonstrate that *FGFR2* is a major cause of syndromic craniosynostosis in the Ghanaian population. However, because genetic testing is not a routine part of clinical care in the study population, the patients had no molecular diagnosis at the time of recruitment. To decipher the genetic etiology of the conditions, we opted for a more exploratory WES over targeted *FGFR2* sequencing for two reasons. Firstly, *FGFR1*, *FGFR2*, and *FGFR3* mutations account for ~75% of syndromic craniosynostosis cases, with overlapping phenotypes. Because of this, targeted sequencing of *FGFR2* alone would have been risky, as there was no genetic test available for the patients. Secondly, because of the variable clinical presentations observed in individuals with the same causative *FGFR2* variant, we hypothesized that additional pathogenic variants may account for phenotypic variability by contributing to co‐occurring syndromes or other phenotypes in the proband. Thus, while *FGFR2* variants remain the primary drivers of the reported craniosynostosis syndromes, we hypothesize that additional pathogenic variants in other genes may culminate in co‐occurring syndromes or other presentations that may account for phenotypes that have been inconsistently and rarely reported in individuals with *FGFR2*‐related syndromes. WES is a more exploratory approach to achieving these two aims.

Several genetic syndromes presenting with craniosynostosis, including Crouzon and Apert syndromes, are frequently caused by GoF mutations in *FGFR2* [1]. These mutations occur particularly within the extracellular immunoglobulin‐like II and III domains, as well as the IgII–III linker domains. The role of these domains in ligand binding and receptor dimerization is critical, as mutations in these regions often destabilize autoinhibitory conformations [41]. Genetic analyses of DNA samples from our cohort revealed six unique likely pathogenic or pathogenic *FGFR2* variants, including heterozygous p.Asp336Gly, p.Cys342Ser, p.Pro253Arg, p.Cys342Gly, p.Gly272del, and p.Ser252Trp in eight families (Table 2). All these variants occurred in the extracellular domain of the FGFR2 receptor. The p.Ser252Trp variant occurred in three probands from different families (Figure 2A). These variants were de novo in five families (Families 1, 2, 3, 5, and 6); however, the unavailability of paternal samples in three families (Families 4, 7, and 8) prevented us from determining the same for these families. Notably, these variants are classified as likely pathogenic or pathogenic by ClinVar and LOVD (Supporting Information 7: Table S3). Moreover, pathogenicity predictive tools (Supporting Information 6: Table S2) and structural modeling of mutant proteins by HOPE (Supporting Information 6: Table S2) supported the pathogenicity of these variants. Apart from the p.Ser252Trp variant that affected all suture types (i.e., metopic, coronal, sagittal, and lambdoid sutures), in the probands, the other five variants affected a subset of sutures (Figure 2B). These variants are localized within the extracellular IgII, IgII–III linker, and IgIII domain (Figure 2C), which are mutational hotspots associated with aberrant osteogenesis and premature suture fusion [41].

All four probands clinically diagnosed with Crouzon syndrome had distinct *FGFR2* pathogenic variants (Table 2), demonstrating the allelic heterogeneity of this syndrome in the study population. These variants included p.Asp336Gly, p.Cys342Ser, p.Cys342Gly, and p.Gly272del. The p.Asp336Gly variant in *FGFR2*, identified in Family 1, was observed in a 12‐year‐old female proband diagnosed with Crouzon syndrome. The proband presented with bicoronal and sagittal craniosynostosis, severe midface hypoplasia (manifesting as a retruded forehead and flattened zygomatic arches), ocular proptosis, and exophthalmos. The Asp336Gly variant occurs in the Ig‐IIIc domain of FGFR2 [3], and substitutions at this residue may destabilize the receptor conformation, thereby altering ligand affinity. Similar mutations in the Ig‐IIIc domain, such as p.Asp321Gly, have been linked to Pfeiffer syndrome, emphasizing the functional importance of this residue in maintaining receptor‐binding affinity [3]. Notably, the Asp336Gly variant has been documented in previous studies as a causative factor for craniosynostosis [42], which resonates with the condition identified in the current proband.

Two unique variants were observed at amino acid residue 342 of FGFR2 in two unrelated probands. The p.Cys342Ser variant was identified in a male proband diagnosed with Crouzon syndrome in Family 2. Moreover, another male proband in Family 4, also clinically diagnosed with Crouzon syndrome, harbored the p.Cys342Gly variant in FGFR2. However, there was phenotypic variability among these two probands. This highlights variable expressivity even among mutations at the same wildtype amino acid residue, although the mutant residues differ. Cys342 is a conserved residue critical for disulfide bond formation within the IgIII domain of FGFR2 [3]. Disruption of this bond, as seen in p.Cys342Ser and p.Cys342Gly variants, may destabilize the receptor structure, permitting constitutive dimerization and hyperactivation of downstream pathways (Supporting Information 9: Table S5). This variability may reflect differences in residual receptor function or interactions with genetic modifiers. In essence, Cys342 substitution has been well documented to be associated with craniosynostosis syndrome [3].

A female proband (Family 5) harbored a heterozygous, de novo p.Gly272del variant in *FGFR2*. This proband was diagnosed with Crouzon syndrome with premature fusion of the sagittal and coronal sutures and proptosis. ClinVar classifies this variant as likely pathogenic (Variation ID: 2780194), and previous studies have documented it as pathogenic for Crouzon syndrome [43, 44]. Moreover, given its location within the Ig‐like C2‐type 3 domain of FGFR2 (Figure 2C), this variant could account for the observed phenotypes. Importantly, VarSome also predicts this variant to be likely pathogenic (Supporting Information 2: Figure S2). In UniProt (https://www.uniprot.org/uniprotkb/P21802/entry, accessed on April 30, 2026), similar in‐frame deletions have been reported in patients with Crouzon, Saethre–Chotzen, and Pfeiffer syndromes. The Crouzon syndrome in‐frame deletion occurred at amino acid Positions 287–289 and 356–358, the Saethre–Chotzen syndrome case at amino acid Positions 269–270, and the Pfeiffer syndrome case at Position 273. Plausibly, these in‐frame deletions may act through a GoF mechanism rather than a loss‐of‐function (LoF) mechanism.

The four probands clinically diagnosed with Apert syndrome harbored the p.Pro253Arg and p.Ser252Trp variants in FGFR2 (Table 2). However, 75% (*n* = 3) of these four probands harbored the p.Ser252Trp variant (Figure 2A). A male proband in Family 3 harbored the p.Pro253Arg variant and presented with premature synostosis of the bicoronal and sagittal sutures. The proband presented with additional features, including severe syndactyly of the hands and feet, high‐arched palate, and intracranial hypertension. Pro253Arg is a canonical Apert syndrome mutation that alters FGFR2 ligand‐binding specificity [45], enhancing its affinity for different FGF ligands. This dysregulated signaling due to the Pro253Arg variant may disrupt cranial suture homeostasis. In harmony with existing literature [12], which indicates that the p.Ser252Trp variant accounts for the majority (~66%) of Apert syndrome cases, we observed this variant in 75% of our probands (Families 6, 7, and 8). Probands exhibited bicoronal or unicoronal craniosynostosis, complex syndactyly (except the thumbs and great toes), and palatal abnormalities. Interestingly, both p.Ser252Trp and p.Pro253Arg variants are located in the IgII–IgIII linker domain (Figure 2C). The Ser252Trp variant increases FGFR2 binding affinity for FGF1, FGF2, and FGF10, thereby promoting ectopic signaling in CNCCs and limb mesenchyme [46]. The lack of complete harmony among the affected sutures in the three probands may reflect modifiers that influence phenotypic expressivity.

The results from our cohort reinforce clear genotype–phenotype correlations in *FGFR2*‐related craniosynostosis, in harmony with existing literature [10]. Crouzon‐associated variants (p.Cys342Ser, p.Cys342Gly, p.Gly272del, and p.Asp336Gly) mainly drive midface craniofacial defects. However, Apert‐associated mutations (p.Pro253Arg and p.Ser252Trp) cause craniofacial defects and limb anomalies, as reported elsewhere [10]. This dichotomy reflects differences in ligand‐binding specificity due to these mutations. Functional studies of *FGFR2* variants using in vitro models confirmed their GoF nature [1]. The p.Ser252Trp variant, for instance, enhances FGFR2 phosphorylation and MAPK/ERK signaling, thereby accelerating osteoblast differentiation. Similarly, p.Cys342 mutations exhibit ligand‐independent receptor dimerization in coimmunoprecipitation assays, confirming their constitutive activity [10]. These findings align with the ClinVar and LOVD pathogenic classifications and support the use of FGFR inhibitors in preclinical models to mitigate craniosynostosis progression [47]. The de novo nature of these variants reduces recurrence risk in subsequent pregnancies, though germline mosaicism remains a concern, yielding a ~1% recurrence risk [48].

A major observation in genetic and genomic studies on *FGFR2*‐related syndromes is the phenotypic variability (especially inconsistently and rarely reported phenotypes) among individuals harboring the same mutation. This was observed in the three probands in our cohort harboring the p.Ser252Trp variant and in previous studies [13, 49]. For example, monozygotic twins with the same p.Ser252Trp variant demonstrated variable expressivity. Whereas both shared classical Apert syndrome phenotypes (craniosynostosis with brachycephaly, hypertelorism, down‐slanting palpebral fissures, micrognathia, midface hypoplasia, and finger and toe syndactyly), one of the twins additionally presented with an inguinal left diaphragmatic hernia and a cleft palate [50]. Furthermore, eight patients, all belonging to a large multiplex pedigree and harboring the same cosegregating p.Cys278Phe variant in *FGFR2*, had variable phenotypes of hearing, vision, mouth, teeth, and head, though they shared classical Crouzon syndrome phenotypes [51]. Lastly, six unrelated patients diagnosed with Apert syndrome harbored the same p.Pro253Arg variant in *FGFR2*, yet had additional variable phenotypes, apart from the shared classical phenotypes. For example, congenital heart disease and bilateral talipes equinovarus occurred in ~33% and ~17% of patients, respectively [52]. Although the age at clinical evaluation may influence these observations, we hypothesized that additional likely pathogenic variants (that may be linked to co‐occurring syndromes or other phenotypes) in the probands may account for the phenotypic variability observed among probands with the same variant. Importantly, a study [36] has shown that patients may harbor more than one pathogenic variant, leading to co‐occurring genetic syndromes with overlapping phenotypes. Moreover, using a WES dataset from diverse populations, analyzed through Genomics England′s PanelApp and OMIM, a study [53] demonstrated that, on average, individuals are born with 4.7 pathogenic or likely pathogenic variants, of which 1.66 are compatible with a Mendelian condition at the genotype level. Thus, individuals may carry more than one pathogenic or likely pathogenic variant concurrently, leading to co‐occurring syndromes or phenotypes whose diverse phenotypes may be misconstrued as those of a single syndrome during clinical evaluation. Corollary to this, we identified additional likely pathogenic variants in genes with craniofacial, neurodevelopmental, and sensory (e.g., hearing) phenotypes that have been reported inconsistently in patients harboring the same *FGFR2* variants (Table 3; Supporting Information 6: Table S2, Supporting Information 7: Table S3, and Supporting Information 8: Table S4). Although these genes are not enriched in any molecular or biological processes, they are enriched in phenotypes reported in individuals with *FGFR2* syndromes (Figure 3). These include “conductive hearing impairment,” “abnormal optic disk morphology,” and “abnormal bone morphology.” These observations are consistent with previous studies demonstrating that genes with divergent molecular and biological functions may direct convergent phenotypes [54]. Interestingly, these genes are expressed at critical developmental time points, cell lineages, and craniofacial structures in mouse (Figure 4) and human embryonic craniofacial tissues (Figure 5). As an example, we observed a pathogenic variant (p.Arg32Cys) in *GJB2* in the proband of Family 4 (Supporting Information 7: Table S3), who was diagnosed with Crouzon syndrome. This variant or gene has been linked to both autosomal dominant and recessive hearing loss in ClinVar, OMIM, and elsewhere [55]. It is also associated with hypertelorism, global developmental delay, intellectual disability, trigonocephaly, brachycephaly, and microcephaly in DECIPHER (Supporting Information 8: Table S4), phenotypes reported in individuals with Crouzon syndrome. Unfortunately, many associated phenotypes (e.g., hearing loss and global developmental delay) were not evident in our proband, likely due to the timing of the clinical evaluation (~1 month old). The proband may develop this phenotype later in life. Moreover, we observed a likely pathogenic variant (p.Asn757His) in *ARVCF*, a gene within the 22q deletion syndrome locus, which is linked to many craniofacial phenotypes in DECIPHER. These include sagittal craniosynostosis, mandibular prognathia, brachycephaly, plagiocephaly, microcephaly, abnormal dental morphology, and bilateral microphthalmos (Supporting Information 8: Table S4). Lastly, we observed a novel variant (p.His337Asn) in *CRISPLD2*, which all variant effect predictors predicted to be pathogenic (Table 3). Variants in *CRISPLD2* are associated with nonsyndromic OFCs [56–58], making it a crucial gene for craniofacial development and phenotypes. Other variants in the same gene have also been associated with asthma and lung function [59, 60], making it crucial for airway function, which may be impaired (e.g., obstructive sleep apnea) in some cases of Apert and Crouzon syndromes [61]. Although the *CRISPLD2* variant observed in this study was inherited from the mother, this is expected of a modifier variant or gene that may increase susceptibility to particular phenotypes by demonstrating variable penetrance and expressivity.

The results of our study must be interpreted in light of some limitations. First, clinical presentations of probands with the same syndrome or mutations were inconsistent. This could be attributed to three factors: (a) Some phenotypes, such as neurodevelopmental disorders (developmental delay and/or intellectual disability, especially in Apert syndrome), increased intracranial pressure, hydrocephalus, additional suture fusion, obstructive sleep apnea, malocclusion, proptosis, and midface retrusion, become more obvious with age [62–66], so the varied age at which the probands were recruited may affect observed phenotypes; (b) individuals with the same *FGFR1*, *FGFR2*, and *FGFR3* syndromes or mutations may show variable penetrance and expressivity, as well as a weak genotype–phenotype correlation—this includes cases of Crouzon, Pfeiffer, and Muenke syndromes; therefore, our reported probands may not necessarily have the same clinical presentations [11, 13, 49, 67]; and (c) our inability to recontact patients to collect phenotypes that may have become apparent later. Second, our assertion about the possible roles of modifier variants (Table 3) should be interpreted with caution, as additional functional experiments are warranted to tease out the relative contributions of these variants to the observed phenotype. For this reason, functional studies in animal, organoid, or cell models, such as CRISPR/Cas‐mediated functional assays, with co‐occurring mutations in *FGFR2* and hypothetical modifier genes are warranted to elucidate the relative contributions of these variants to the observed phenotypes. Relatedly, the variants reported in Table 3 must be interpreted in the context of the inheritance patterns of the diseases associated with the genes harboring these variants. For example, the novel *HSPG2* variant (p.Leu4242Arg) must be interpreted with care, as this gene is currently associated with autosomal recessive syndromes in OMIM, but the variant we report is heterozygous. Third, WES is not designed to capture noncoding variants, so our study may have missed variants with regulatory functions, as well as structural variants. Irrespective of these limitations, our study demonstrates that WES is a more exploratory approach to identifying primary or cardinal mutations underlying genetic syndromes such as Apert and Crouzon, as well as potential modifiers.

## 5. Conclusion

Our crucial genetic investigation into *FGFR2*‐related craniosynostosis in a sub‐Saharan African cohort implicated Apert and Crouzon syndromes as cardinal syndromic causes of craniosynostosis. More diverse pathogenic alleles underlie Crouzon syndrome, whereas two alleles drive Apert syndrome, with ~75% of cases harboring the p.Ser252Trp variant. This study addresses a critical knowledge gap in African populations, which are underrepresented in omics research. This study characterizes the mutational spectrum of *FGFR2*‐related craniosynostosis in our cohort and emphasizes the importance of population‐specific genetic studies. Moreover, WES has proven to be a better approach for identifying primary driver mutations in genetic conditions, as well as variants that may contribute to phenotypic variability. These observations have important implications for disease etiopathology, genetic counseling (recurrent risk estimation and prenatal testing), molecular diagnosis, and other clinical management of craniosynostosis. It also contributes to the global effort to achieve health equity by ensuring inclusive genetic research among historically underrepresented populations.

## Author Contributions

L.J.J.G. contributed to conception, design, data acquisition, analysis, and interpretation and drafted and critically revised the manuscript. M.D.A. contributed to design, data acquisition, analysis, and interpretation and drafted and critically revised the manuscript. D.K.S., G.O.M., B.T., C.O.A., J.B.D., S.O‐Y., T.B., A.B., P.D., and M.L.C. contributed to design, data acquisition, analysis, and interpretation and critically revised the manuscript.

## Funding

This project was supported by the Fogarty International Center (FIC) at the National Institutes of Health (NIH), United States (Grant Number: R21TW011729; L.J.J.G., S.O.Y., and M.L.C.).

## Disclosure

All authors gave their final approval and agreed to be accountable for all aspects of the work.

## Ethics Statement

This study was approved by the Institutional Review Board (IRB) at KATH (KATH‐IRB/AP/032/20), Kumasi, Ghana. Written informed consent was obtained from all participating families. The study was conducted in line with the Declaration of Helsinki.

## Conflicts of Interest

The authors declare no conflicts of interest.

## Supporting Information

Additional supporting information can be found online in the Supporting Information section.

## Supporting information


**Supporting Information 1** Figure S1: The pedigrees of the eight families recruited for the study are shown. Affected individuals in each family are shaded. Individuals from whom samples were obtained are marked with C or S, where C denotes a cheek swab and S denotes saliva. The mode of inheritance for each pedigree is also indicated below. The genotype of each FGFR2 variant for each family is also indicated for all individuals from whom samples were obtained.

**Supporting Information 2** Figure S2: This figure is a screenshot of the detailed VarSome prediction for the p.Gly272del FGFR2 variant. It shows that the variant is “likely pathogenic,” with a phyloP100 score of 7.899.

**Supporting Information 3** Figure S3: The chromatogram from Sanger sequencing, used as orthogonal validation of the p.Gly272del FGFR2 variant, is shown here. Panels to the left show forward‐directional sequencing, whereas those to the right depict reverse‐directional sequencing. Thus, the variant was confirmed in both directions.

**Supporting Information 4** Figure S4: This figure presents a detailed output of the enriched pathways from g:Profiler. It shows that the implicated genes are enriched for craniofacial development and phenotypes.

**Supporting Information 5** Table S1: The thresholds for the various pathogenicity predictive tools employed in the study are shown in this table. These include tools for missense variants and loss‐of‐function variants.

**Supporting Information 6** Table S2: The table presents pathogenicity predictions from 11 tools used to assess missense variants. It is color‐coded to facilitate the easy identification of pathogenic (red) and benign (green) predictions. It also includes the CADD prediction for a nonsense mutation. Lastly, it contains frameshift variants, for which no available tool could effectively predict their pathogenicity.

**Supporting Information 7** Table S3: This table presents further evaluation of the implicated variants by assessing whether they have been classified in various databases and by reporting their minor allele frequencies. Further pathogenicity assessment used ClinVar and LOVD, whereas minor allele frequency checks used the African Genome Variation Database (AGVD) and gnomAD.

**Supporting Information 8** Table S4: To assess the relevance of the genes harboring the implicated variants, this table summarizes reported phenotypes associated with these genes. These phenotypes were curated from databases including DECIPHER, Online Mendelian Inheritance in Man (OMIM), Mouse Genome Informatics (MGI), and Alliance of Genome Resources (AGR).

**Supporting Information 9** Table S5: This table presents the results of the in silico simulation of the effects of reported variants on protein structure and function, using the HOPE tool. It assesses the effects of the implicated variants on protein structure by comparing wildtype and mutant residues across size, charge, hydrophobicity, and conservation.

## Data Availability

The whole exome sequencing dataset reported in this article is available through the FaceBase Consortium (https://www.facebase.org) under Accession Number B1‐TQ60 (Gowans, 2026). The data and materials that support the findings of this study are available from the corresponding author upon reasonable request.
