# Supplementary material for: Spectrum of FGFR2 Mutations and Plausible Phenotypic Modifiers Contributing to the Etiology of Syndromic Craniosynostosis in Sub‐Saharan Africans
Source: Int J Genomics. 2026 Nov 11;2026:5753150. doi: 10.1155/ijog/5753150 (PMC13752804; doi:10.1155/ijog/5753150)
Supplement: Supplementary file 1 [file IJOG-2026-5753150-s001.docx]

**Figure S3**
