# Supplementary material for: Spectrum of FGFR2 Mutations and Plausible Phenotypic Modifiers Contributing to the Etiology of Syndromic Craniosynostosis in Sub‐Saharan Africans
Source: Int J Genomics. 2026 Nov 11;2026:5753150. doi: 10.1155/ijog/5753150 (PMC13752804; doi:10.1155/ijog/5753150)
Supplement: Supplementary file 4 [file IJOG-2026-5753150-s004.docx]

**Table S3**

| **Family ID** | **Genomic coordniate** | **Gene** | **HGVSc** | **HGVSp** | **ClinVar** | **LOVD** | **AGVD SSAF MAF** | **gnomAD (Afr/AfrAmr MAF)** | **gnomAD (Global MAF)** | **ACMG/ AMP Codes** |
| --- | --- | --- | --- | --- | --- | --- | --- | --- | --- | --- |
| Family 1 | Chr10:121517396 (rs1057519042) | *FGFR2* | c.1007A>G | p.Asp336Gly | Pathogenic/Likely pathogenic | Likely pathogenic | N/A | N/A | N/A | PS1, PS2, PS3, PS4, PM1, PM2, PP2, PP3, PP4 |
|  | Chr22:19973288  (rs758305598) | *ARVCF* | c.2269A>C | p.Asn757His | N/A | N/A | N/A | 0.0001202 | 0.000005605 | PS2, PM2, PP3 |
|  | Chr1:1645163 (Novel) | *CDK11B* | c.593_594insGGAGG | p.Arg202SerfsTer19 | N/A | N/A | N/A | 0.007333 | 0.00007806 | ^*^PVS1_Strong, PS2, PM2 |
|  | Chr3:184971748 (rs758939120) | *VPS8* | c.3416G>A | p.Arg1139His | N/A | N/A | N/A | 0.00005343 | 0.00001737 | PM2, PM3, PP3 |
| Family 2 | Chr10:121517378 (rs121918487) | *FGFR2* | c.1025G>C | p.Cys342Ser | Pathogenic | Likely pathogenic | N/A | N/A | N/A | PS1, PS2, PS3, PS4, PM1, PM2, PP2, PP3, PP4 |
|  | Chr16:84873019 (Novel) | *CRISPLD2* | c.1009C>A | p.His337Asn | N/A | N/A | N/A | 0.00002667 | 0.000001239 | PM2, PP3 |
| Family 3 | Chr10:121520160 (rs77543610) | *FGFR2* | c.758C>G | p.Pro253Arg | Pathogenic/Likely pathogenic | Pathogenic | N/A | N/A | N/A | PS1, PS2, PS3, PS4, PM1, PM2, PP2, PP3, PP4 |
|  | Chr14:104945000 (rs779995601) | *AHNAK2* | c.10450_10451insTACA | p.Gly3484ValfsTer38 | N/A | N/A | N/A | N/A | N/A | ^*^PVS1_Strong, PS2, PM2 |
|  | Chr15:41866454 (rs974936065) | *SPTBN5*^#^ | c.6520G>T | p.Glu2174Ter | N/A | N/A | N/A | 0.00001349 | 6.26E-07 | ^*^PVS1_Strong, PM2, PM3 |
|  | Chr15:41879329 (Novel) |  | c.3113T>C | p.Leu1038Pro | N/A | N/A | N/A | N/A | N/A | PM2, PM3, PP3 |
| Family 4 | Chr10:121517379 (rs121918488) | *FGFR2* | c.1024T>G | p.Cys342Gly | Pathogenic | Likely pathogenic | N/A | N/A | N/A | PS1, PS2, PS3, PS4, PM1, PM2, PP2, PP3, PP4 |
|  | Chr1:21824556 (Novel) | *HSPG2* | c.12725T>G | p.Leu4242Arg | N/A | N/A | N/A | N/A | N/A | PM2, PP3 |
|  | Chr13:20189488 (rs371024165) | *GJB2* | c.94C>T | p.Arg32Cys | Pathogenic | N/A | N/A | 0.0003604 | **0.00003532** | PS1, PS3, PS4, PM1, PM2, PP2, PP3 |
|  | Chr17:82941446 (Novel) | *TBCD* | c.3527A>C | p.Asp1176Ala | N/A | N/A | N/A | N/A | N/A | PM2, PP2, PP3 |
|  | Chr22:46974368 (rs1333784593) | *TBC1D22A* | c.1094G>C | p.Trp365Ser | N/A | N/A | N/A | 0.0000267 | **0.000001241** | PM2, PP3 |
|  | Chr6:109162950 (Novel) | *CEP57L1* | c.1366_1367dup | p.Met457SerfsTer14 | N/A | N/A | N/A | N/A | N/A | ^*,#^PVS1_Strong, PM2 |
|  | Chr14:104951131 (rs531942140) | *AHNAK2* | c.4319_4320insG | p.Asp1442ArgfsTer10 | N/A | N/A | N/A | 0.001352 | **0.0001053** | ^*^PVS1_Strong, PS2, PM2 |
| Family 5 | Chr10:121520100 | *FGFR2* | c.815_817del | p.Gly272del | N/A | N/A | N/A | N/A | **N/A** | PS1, PS2, PS4, PM1, PM2, PM4, PP3, PP4 |
|  | Chr7:102483656 (rs1554581975) | *RASA4B* | c.2348A>G | p.His783Arg | N/A | N/A | N/A | N/A | N/A | PS2, PM2, PP3 |
|  | Chr14:104948679 (Novel) | *AHNAK2* | c.6771_6772insGG | p.Ile2258GlyfsTer9 | N/A | N/A | N/A | N/A | N/A | ^*^PVS1_Strong, PS2, PM2 |
|  | Chr14:104948682 (rs1215492197) |  | c.6765_6768del | p.Ile2256ThrfsTer9 | N/A | N/A | N/A | N/A | N/A | ^*^PVS1_Strong, PS2, PM2 |
|  | Chr11:111875482 (rs1242596116) | *FDXACB1* | c.1314del | p.Lys438AsnfsTer12 | N/A | N/A | N/A | 0.0001478 | **0.000007441** | ^*^PVS1_Strong, PM2 |
|  | Chr11:111875813 (Novel) |  | c.983del | p.Gly328GlufsTer25 | N/A | N/A | N/A | N/A | N/A | ^*^PVS1_Strong, PM2 |
| Family 6 | Chr10:121520163 (rs79184941) | *FGFR2* | c.755C>G | p.Ser252Trp | Pathogenic | Pathogenic | N/A | N/A | N/A | PS1, PS2, PS3, PS4, PM1, PM2, PP2, PP3, PP4 |
|  | Chr8:93772596 (Novel) | *TMEM67* | c.659C>A | p.Ser220Tyr | N/A | N/A | N/A | N/A | N/A | PS2, PM2, PP3 |
| Family 7 | Chr10:121520163 (rs79184941) | *FGFR2* | c.755C>G | p.Ser252Trp | Pathogenic | Pathogenic | N/A | N/A | N/A | PS1, PS2, PS3, PS4, PM1, PM2, PP2, PP3, PP4 |
|  | Chr19:12661286 (Novel) | *MAN2B1* | c.1000A>G | p.Lys334Glu | N/A | N/A | N/A | N/A | N/A | PM2, PP2, PP3 |
| Family 8 | Chr10:121520163 (rs79184941) | *FGFR2* | c.755C>G | p.Ser252Trp | Pathogenic | Pathogenic | N/A | N/A | N/A | PS1, PS2, PS3, PS4, PM1, PM2, PP2, PP3, PP4 |
|  | Chr1:149464092 (rs1306186424) | *NOTCH2NLC* | c.823del | p.Thr275GlnfsTer29 | N/A | N/A | N/A | 0.004546 | **0.0003722** | ^*,#^PVS1_Strong, PM2 |

The ACMG/AMP code is based on Table 3 in Richards et al. (2015). This was supplemented by ClinGene Sequence Variant Interpretation (SVI) guideline (https://clinicalgenome.org/tools/clingen-variant-classification-guidance/). **^*^**ClinGene Sequence Variant Interpretation (SVI) guideline, as the ACMG criteria for such variants do not allow for new genes. ^#^Variant extends protein length beyond the 460 amino acid residues in UniProt (<https://www.uniprot.org/uniprotkb/Q8IYX8/entry>). PVS1_Strong: ClinGene SVI evidence based on the sequencial decision tree (a) mutant mRNA not predicted to undergo nonsense-mediated decay, (b) Role of region in protein function is unknown, (c) LoF variants in this exon are not frequent in the general population and exon is present in biologically relevant transcript(s), and (d) Variant removes >10% of protein. PS1: Same amino acid change as a previously established pathogenic variant, regardless of nucleotide change. PS2: *De novo* (both maternity and paternity confirmed) in a patient with the disease and no family history. PS3: Well-established *in vitro* or *in vivo* functional studies supportive of a damaging effect on the gene or gene product. PS4: The prevalence of the variant in affected individuals is significantly increased compared to the prevalence in controls. PM1: Located in a mutational hot spot and/or critical and well-established functional domain (e.g. active site of an enzyme) without benign variation. PM2: Absent from controls (or at extremely low frequency if recessive) (see Table 6) in Exome Sequencing Project, 1000 Genomes or ExAC. PM3: For recessive disorders, detected in trans with a pathogenic variant. PM4: Protein length changes due to in-frame deletions/insertions in a non-repeat region or stop-loss variants. PP2: Missense variant in a gene that has a low rate of benign missense variation and where missense variants are a common mechanism of disease. PP3: Multiple lines of computational evidence support a deleterious effect on the gene or gene product (conservation, evolutionary, splicing impact, etc.). PP4: Patient’s phenotype or family history is highly specific for a disease with a single genetic etiology.
