# Supplementary material for: Spectrum of FGFR2 Mutations and Plausible Phenotypic Modifiers Contributing to the Etiology of Syndromic Craniosynostosis in Sub‐Saharan Africans
Source: Int J Genomics. 2026 Nov 11;2026:5753150. doi: 10.1155/ijog/5753150 (PMC13752804; doi:10.1155/ijog/5753150)
Supplement: Supplementary file 5 [file IJOG-2026-5753150-s005.docx]

**Table S4**

| **Gene** | **DECIPHER** | **Online Mendelian Inheritance in Men (OMIM)** | **Mouse Genome Informatics (MGI** | **Alliance of Genome resources (AGR)** |  |
| --- | --- | --- | --- | --- | --- |
| *FGFR2* | Brachycephaly, Intellectual disability, Low-set ears, Prominent nasal bridge, Finger clinodactyly, Hypertelorism, Downslanted palpebral fissures, High palate, Microcephaly, Abnormal pinna morphology, Thin upper lip vermilion, Frontal bossing, Microtia, Prominent nose, Upslanted palpebral fissure, Cleft palate, Depressed nasal bridge, Global developmental delay, Malar flattening, 2-3 toe syndactyly, Brachydactyly, Deeply set eye, Dolichocephaly, Facial asymmetry, Delayed speech and language development, Mandibular prognathia, Plagiocephaly, Protruding ear, Ptosis, Abnormality of the outer ear, Hearing impairment, Bifid uvula, Conductive hearing impairment, Midface retrusion, Mild intellectual disability, Sparse hair, Trigonocephaly | Apert syndrome (AD), Crouzon syndrome (AD), Scaphocephaly, maxillary retrusion, and impaired intellectual development, Antley-Bixler syndrome without genital anomalies or disordered steroidogenesis (AD), Beare-Stevenson cutis gyrata syndrome (AD), Bent bone dysplasia syndrome (AD), Craniofacial-skeletal-dermatologic dysplasia (AD), Craniosynostosis (nonspecific), Gastric cancer (somatic), Jackson-Weiss syndrome (AD), LADD syndrome 1 (AD), Pfeiffer syndrome (AD), Saethre-Chotzen syndrome (AD), Scaphocephaly and Axenfeld-Rieger anomaly | Abnormal coronal suture morphology, exophthalmos, ocular hypertelorism, premature coronal suture closure, short face, shortened head, decreased limb number, abnormal chondrocyte morphology, abnormal craniofacial morphology, abnormal ear development, abnormal endochondral bone ossification, abnormal palatal shelf fusion at midline, cleft secondary palate, decreased cranium height, domed cranium, malocclusion, abnormal cranial cartilage morphology, abnormal cranium morphology, abnormal ear development, abnormal limb development | **Zebrafish Phenotype:** cell proliferation in midbrain decreased occurrence (abnormal), ethmoid cartilage decreased length (abnormal), optic tectum physical object quality (abnormal), **Human Disease association**: craniosynostosis, Crouzon syndrome, acrocephalosyndactylia, lacrimoauriculodentodigital syndrome 1, Antley-Bixler syndrome without disordered steroidogenesis, Beare-Stevenson cutis gyrata syndrome, Antley-Bixler syndrome, bent bone dysplasia syndrome 1, Jackson-Weiss syndrome, Pfeiffer syndrome, Saethre-Chotzen syndrome, bone disease, Antley-Bixler syndrome with disordered steroidogenesis, |  |
| *ARVCF* | Sagittal craniosynostosis, Mandibular Prognathia, Brachycephaly, Plagiocephaly, Microcephaly, Abnormal dental morphology, Bilateral microphthalmos | N/A | Decreased bone mineral density | **Zebrafish Phenotype** — Abnormal skull morphology, Abnormal facial shape, Multiple sutures craniosynostosis, Turricephaly **Human Disease Association** — DiGeorge syndrome (AD) |  |
| *CDK11B* | Hypertelorism, Abnormality of the eye, Deep-set eyes, Microcephaly, Brachycephaly, Plagiocephaly, Small forehead, Midface retrusion, Mandibular Prognathia, Short chin | N/A | N/A | **Zebrafish Phenotype** — N/A **Human Disease Association** — N/A |  |
| *VPS8* | Craniosynostosis, Microcephaly, Plagiocephaly, Trigonocephaly, Deep-set eye, Hypertelorism, Dental malocclusion, Craniofacial asymmetry, Short chin, Bilateral microphthalmos | N/A | N/A | **Zebrafish Phenotype** — N/A **Human Disease Association** — N/A |  |
| *CRISPLD2* | Sleep apnea, High palate, Proptosis, Respiratory failure, Hypoplasia of the maxilla, Microcephaly, Dolichocephaly | N/A | Decreased bone mineral density | **Zebrafish Phenotype** — N/A **Human Disease Association** — Orofacial cleft (AD) (*Danio rerio*) |  |
| *AHNAK2* | High palate, 2-3 toe syndactyly, Microcephaly, Dolichocephaly, Turricephaly, Pleagiocephaly, Brachycephaly, Craniosynostosis | N/A | N/A | **Zebrafish Phenotype** — N/A **Human Disease Association** — N/A |  |
| *SPTBN5*^#^ | Craniosynostosis, Brachycephaly, Microcephaly, Turricephaly | N/A | N/A | **Zebrafish Phenotype** — N/A **Human Disease Association** — N/A |  |
|  |  |  |  |  |  |
|  |  |  |  |  |  |
| *HSPG2* | Microcephaly, Midface retrusion | Dyssegmental dysplasia, Silverman-Handmaker type (AR) Schwartz-Jampel syndrome, type 1 (AR) | Abnormal craniofacial morphology, Abnormal cranial morphology, Exencephaly, Flat face | **Zebrafish Phenotype** — Brachycephaly, Midface retrusion, Microcephaly, Proptosis **Human Disease Association** — Schwartz-Jampel syndrome 1 (AR), Silverman - Handmaker type dyssegmental dysplasia (AR), Osteochondrodysplasia, Carotid artery disease, |  |
| *GJB2* | hypertelorism, global developmental delay, Intellectual disability, trigonocephaly, brachycephaly, microcephaly | Bart-Pumphrey syndrome (AD) Deafness, autosomal dominant 3A (AD) Deafness, autosomal recessive 1A (AD)(DD) Vohwinkel syndrome (AD) Keratitis-ichthyosis-deafness syndrome (AD) | Abnormal eye morphology | **Zebrafish Phenotye** — Abnormal facial shape **Human Disease Association** — Bart-Pumphrey syndrome (AD), Keratitis-ichthyosis-deafness syndrome (AD), Nonsyndromic deafness 3A (AD), Nonsyndromic deafness 1A (AR), Vohwinkel syndrome (AD) |  |
| *TBCD* | Scaphocephaly, Brachycephaly, Plagiocephaly, Dolichocephaly, Trigonocephaly, Microcephaly, Turricephaly, Lissencephaly, Midface retrusion | Encephalopathy, progressive, early-onset, with brain atrophy and thin corpus callosum (AR) | N/A | **Zebrafish Phenotype** — N/A  **Human Disease Association**: Microcephaly, Plagiocephaly, early onset progressive encephalopathy with brain atrophy and thin corpus callosum (AR) |  |
| *TBC1D22A* | Microcephaly, Plagiocephaly, Dolichocephaly, Brachycephaly, Holoprosencephaly, Lissencephaly, Trigonocephaly, | N/A | N/A | **Zebrafish Phenotype** — N/A **Human Disease Association** — N/A |  |
| *CEP57L1* | Brachycephaly, Microcephaly, Plagiocephaly, Short chin | N/A | N/A | **Zebrafish Phenotype** — N/A **Human Disease Association** — N/A |  |
| *RASA4B* | Microcephaly, Brachycephaly, Plagiocephaly, Anencephaly, Proptosis | N/A | N/A | **Zebrafish Phenotype** — N/A **Human Disease Association** — N/A |  |
| *FDXACB1* | Craniosynostosis, Proptosis, Dolichocephaly, Microcephaly, Trigonocephaly, Brachycephaly, Plagiocephaly, Turricephaly, Holopreosencephaly | N/A | Decreased bone mineral density | **Zebrafish Phenotype** — N/A **Human Disease Association** — N/A |  |
|  |  |  |  |  |  |
| *TMEM67* | 2-4 toes cutaneous syndactyly, Proptosis, High palate, Low-set ears, Microcephaly, Dolichocephaly, Turricephaly | COACH syndrome 1 (AR) Joubert syndrome 6 (AR) Meckel syndrome 3 (AR) Nephronophthisis 11 (AR) | Microcephaly, Exencephaly | **Zebrafish Phenotype** — Anencephaly, Cutaneous syndactyly, Lobar holoprosencephaly, Low-set ears, Microcephaly, Proptosis **Human Disease Association** — Bardet-Beidl Syndrome (AR), Coach syndrome (AR), Joubert syndrome 6 (AR), Meckel syndrome 3 (AR), Nephrononphthisis (AR) |  |
| *MAN2B1* | Microcephaly, plagiocephaly, Dolichocephaly, High palate | Mannosidosis, alpha-, types I and II (AR) | N/A | **Zebrafish Phenotype** — Brachycephaly, Craniosynostosis, Cutaneous syndactyly, Microcephaly **Human Disease Association** — alpha-mannosidosis (AR) |  |
| *NOTCH2NLC* | Brachycephaly, Microcephaly | Neuronal intranuclear inclusion disease (AD) Oculopharyngodistal myopathy 3 (AD) Tremor, hereditary essential, 6 (AD) | N/A | **Zebrafish Phenotype** — N/A **Human Disease Association** — Essential tremor 6 (AD), Neuronal intranuclear inclusion disease (AD), Oculopharyngodistal myopathy-3 (AD) |  |
