# Supplementary material for: Spectrum of FGFR2 Mutations and Plausible Phenotypic Modifiers Contributing to the Etiology of Syndromic Craniosynostosis in Sub‐Saharan Africans
Source: Int J Genomics. 2026 Nov 11;2026:5753150. doi: 10.1155/ijog/5753150 (PMC13752804; doi:10.1155/ijog/5753150)
Supplement: Supplementary file 6 [file IJOG-2026-5753150-s006.docx]

**Table S1**

| **Tool** | **Range** | **Predictive threshold** |
| --- | --- | --- |
| ClinPred | 0-1 | >0.5 |
| MetaRNN | 0-1 | >0.5 |
| BayesDel_addAF | -1.11707-0.750927 | >0.0692655 |
| REVEL | 0-1 | >0.5 |
| CADD | 0-50+ | >20 |
| AlphaMissense | 0-1 | Pathogenic >=0.564 |
| MutPred | 0-1 | >0.5 |
| PolyPhen2 | 0-1 | >0.5 |
| MutationAssessor | -5.17-6.49 | >=1.935 |
| MutationTaster_rankscore |  | >0.31733 |
| SIFT | 0-1 | <0.05 |
| SpliceAI | 0-1 | 0.20≥ |
