# Supplementary material for: Spectrum of FGFR2 Mutations and Plausible Phenotypic Modifiers Contributing to the Etiology of Syndromic Craniosynostosis in Sub‐Saharan Africans
Source: Int J Genomics. 2026 Nov 11;2026:5753150. doi: 10.1155/ijog/5753150 (PMC13752804; doi:10.1155/ijog/5753150)
Supplement: Supplementary file 9 [file IJOG-2026-5753150-s009.docx]

**Table S5**

| **Mutants** | **Amino Acids Properties and Structural Effect.** | **Amino acid substitutions** | **Domain Effect** |
| --- | --- | --- | --- |
| FGFR2_Ser252Trp | The mutant residue is bigger than the wild-type residue, as this might create bumps. The mutant residue is more hydrophobic than the wild-type residue, which can result in loss of hydrogen bonds and/or disturb correct folding. The wild-type residue is predicted to be located in its preferred secondary structure, a turn. The mutant residue prefers to be in another secondary structure; therefore, the conformation will be slightly destabilized. |   Serine  Tryptophan | The mutated residue is located in a domain (Ig-like Fold) which is involved in a wide variety of functions, which usually requires interaction of the intact domain with another protein/molecule. A mutation of a residue in this domain could disturb this interaction. |
| FGFR2_Asp336Gly | The mutant residue is smaller than the wild-type residue, which will cause a possible loss of external interactions. The wild-type residue charge was negative, the mutant residue charge is neutral, which will disturb the ionic interaction made by the original, wild-type residue. This can cause loss of interactions with other molecules. The mutant residue is more hydrophobic than the wild-type residue, which will affect hydrogen bond formation. Also, the wild-type residue forms a salt bridge with: Lysine at position 272. The difference in charge will disturb the ionic interaction made by the original, wild-type residue. |   Glycine  Aspartate | The mutation lies within the Ig-like C2-type 3 domain. The mutant residue, glycine, introduces high flexibility and disrupts the structural rigidity required for domain stability and function. |
| FGFR2_Cys342Ser | The wild-type residue is more hydrophobic than the mutant residue. The mutation will cause loss of hydrophobic interactions in the core of the protein. The wild-type residue is involved in a cysteine bridge, which is important for stability of the protein. Only cysteines can make these types of bonds. The mutation causes loss of this interaction and will have a severe effect on the 3D-structure of the protein. Together with loss of the cysteine bond, the differences between the old and new residue can cause destabilization of the structure. |   Serine  Cysteine | The mutation is located within the Ig-like C2-type 3 domain. The mutation introduces an amino acid with different properties, which can disturb this domain and abolish its function. |
| FGFR2_Pro253Arg | The wild-type residue charge was neutral, the mutant residue charge is positive. This can cause repulsion between the mutant residue and neighbouring residues. The mutant residue is bigger than the wild-type residue. The residue is involved in a multimer contact, which is highly likely to be biologically relevant. The bigger mutant residue at this position can disturb the multimeric interactions. Prolines are known to be very rigid and therefore induce a special backbone conformation which might berequired at this position. The mutation can disturb this special conformation. |   Arginine  Proline | The mutated residue is situated on the surface of a domain with unknown function and does not contact other domains of established function in the modelled structure. Nevertheless, potential interactions with external molecules or unmodeled domains remain possible and could be disrupted by this mutation. |
| p.Cys342Gly | The wildtype residue (Cysteine) is bigger and more hydrophobic than the mutant residue (glycine). Thus, the mutation may lead loss of hydrophobic and other interactions of the protein. The wildtype residue forms a cysteine bridge, which is essential for protein stability. The loss of this cysteine bridge have severe consequence on the 3D structure of the protein. |   Glycine  Cysteine | The mutant is located within the Ig-like C2-type 3 domain of the protein. The introduction of a more flexible glycine may alter the required rigidity of the protein at this position. The affected domain interacts with ligands and its mutant residue may hamper such interactions. |
